# *Stemodia maritima* L. Extract Decreases Inflammation, Oxidative Stress, and Alveolar Bone Loss in an Experimental Periodontitis Rat Model

**DOI:** 10.3389/fphys.2017.00988

**Published:** 2017-12-01

**Authors:** Alrieta H. Teixeira, Jordânia M. de Oliveira Freire, Luzia H. T. de Sousa, Antônia T. Parente, Nayara A. de Sousa, Angela M. C. Arriaga, Francisca R. Lopes da Silva, Iracema M. Melo, Igor I. Castro da Silva, Karuza M. A. Pereira, Paula Goes, José J. do Nascimento Costa, Gerardo Cristino-Filho, Vicente de Paulo T. Pinto, Hellíada V. Chaves, Mirna M. Bezerra

**Affiliations:** ^1^RENORBIO, Federal University of Ceará, Fortaleza, Brazil; ^2^Dentistry School, Federal University of Ceará, Sobral, Brazil; ^3^Medical School, Federal University of Ceará, Sobral, Brazil; ^4^Department of Organic and Inorganic Chemistry, Federal University of Ceará, Fortaleza, Brazil; ^5^Department of Morphology, Medical School, Federal University of Ceará, Fortaleza, Brazil; ^6^Department of Pathology and Legal Medicine, Medical School, Federal University of Ceará, Fortaleza, Brazil; ^7^UNINTA University Center, Sobral, Brazil

**Keywords:** *Stemodia maritima* L., periodontitis, inflammation, oxidative stress, bone loss

## Abstract

Periodontitis is very prevalent worldwide and is one of the major causes of tooth loss in adults. About 80% of the worldwide population use medicinal plants for their health care. *Stemodia maritima* L. (*S. maritima*) antioxidant and antimicrobial effects *in vitro* as well as anti-inflammatory properties. Herein, the potential therapeutic effect of *S. maritima* was assessed in rats subjected to experimental periodontitis (EP). EP was induced in female Wistar rats by nylon thread ligature around 2nd upper left molars for 11 days. Animals received (*per os*) *S. maritima* (0.2; 1 or 5 mg/kg) or vehicle (saline + DMSO) 1 h before ligature and then once daily for 11 days. The naive group had no manipulation. After this time-point, the animals were terminally anesthetized, and the maxillae were removed for morphometric and histological analyzes (HE). Gingival tissues were dissected to cytokine levels detection (TNF-α, IL1-β, CINC-1, and IL-10), enzymes superoxide dismutase (SOD), and catalase (CAT) analysis, as well as gene expression (TNF-α, IL-1β, RANK, and iNOS) by qRT-PCR. Systemic parameters (weight variation, plasma levels of hepatic enzymes aspartate aminotransferase (AST) and alanine aminotransferase (ALT), creatinine, total alkaline phosphatase (TALP), and bone alkaline phosphatase (BALP) were performed. Histological analysis of the stomach, liver, kidney, and heart was also performed. *S. maritima* (5 mg/kg) decreased alveolar bone loss, TNF-α and CINC-1 gingival levels, oxidative stress, and transcription of TNF-α, IL1-β, RANK, and iNOS genes. It elevated both BALP activity and IL-10 gingival levels. The animals showed no any signs of toxicity. In conclusion, *S. maritima* reduced pro-inflammatory cytokine production, oxidative stress, and alveolar bone loss in a pre-clinical trial of periodontitis. *S. maritima* is a potential tool for controlling the development of periodontitis.

## Introduction

Current concepts define periodontitis as a chronic inflammatory disease that compromises the integrity of the tooth-supporting tissues. It is an imbalance between the polymicrobial biofilm and the immune-inflammatory response and includes genetic and environmental risk factors (Genco and Borgnakke, 2013; Hajishengallis, 2014). The periodontal tissue breakdown is mainly mediated by the release of pro inflammatory cytokines (TNF-α, IL-1, and IL-8/CXCL8 (in humans) or CINC-1/CXCL1 (in rats) as well as the production of reactive oxygen species (ROS) (Liao et al., 2014; Hienz et al., 2015; Silva et al., 2015; Gomes et al., 2016).

Mechanical therapy and surgical procedures have been used to treat periodontitis (Wang et al., 2014). Nevertheless, these procedures are not always satisfactory. Thus, adjunctive therapies may be necessary including antibiotics and non-steroidal anti-inflammatory drugs. The major disadvantage of these agents is the development of bacterial resistance and gastric/renal toxicity. Thus, the search for newer and safer therapeutic agents continues to overcome these limitations. Phytochemicals isolated from plants are considered good alternatives to synthetic chemicals. In recent years, the use of plant extracts has gained popularity (Alviano and Alviano, 2009; Chandra Shekar et al., 2015; Kala et al., 2015; Ramesh et al., 2016) and some of these extracts have been used to treat periodontitis or repair bone defects (Guimarães et al., 2016; Lima et al., 2017; Oliveira et al., 2017).

In this regard, earlier chemical studies on *Stemodia maritima* L. (Plantaginaceae family, formely Scrophulariaceae), which is used in traditional medicine to treat inflammatory disorders, reported the isolation of flavonoids, diterpenes, and other compounds associated with larvicidal and antiviral activities (Rodrigues et al., 2010; Russell et al., 2011). Our group studied the phytochemicals in *S. maritima* L., and described the isolation of the natural compound, stemodinol along with seven known compounds (da Silva et al., 2014). Antioxidant and antimicrobial activities were observed on crenatoside, stemodinoside B, and stemodin. These are natural compounds also derived from *S. maritima* L. (da Silva et al., 2014).

Despite these data, there is no study in the literature focusing on inflammatory bone resorption using *S. maritima* L. extract or its bioactive phytochemicals. Thus, considering the involvement of both polymicrobial biofilm (Genco and Borgnakke, 2013; Hajishengallis, 2014) and oxidative stress (Tóthová et al., 2015; Lima et al., 2017) in periodontitis, and the potential therapeutic effect of *S. maritima* L., the goal of this study was to investigate the efficacy of *S. maritima* L. extract in a rat periodontitis model.

## Materials and methods

### Plant material

*S. maritima* L. (*S. maritima* or Sm) is a common shrub that grows widely in the northeast region of Brazil, near the seacoasts. It belongs to Plantaginaceae family, tribe Gratioleae Benth (Albach et al., 2005). Dr. F. S. Cavalcanti and Prof. E. P. Nunes identified the plant and a voucher specimen (# 38483) was registered at Prisco Bezerra Herbarium, Federal University of Ceará, Fortaleza, Brazil. For this study, the extract was obtained from fresh leaves of Sm collected during the flowering stage along Fleixeiras Beach, Ceará, Brazil (da Silva et al., 2014).

### Animals

The experimental procedures and treatments performed on animals were approved by the Animal Research Ethics Committee of Federal University of Ceará (Permit number: 08/2013) in accordance with the guidelines from the Brazilian Society of Laboratory Animal Science - SBCAL/COBEA). Female Wistar rats, 10-weeks-old and weighting 200 ± 20 g were housed in appropriated cages of polypropylene and maintained on a 12 h-12 h light-dark cycles with a constant room temperature of 25°C and received water and food *ad libitum*.

### Experimental design

For the initial experiment, 30 animals were randomly divided into five specific groups (*n* = 6 in each group): unchallenged group (naive), three experimental periodontitis-challenged groups (EP) receiving oral gavage (*per os*) in different concentrations of *S. maritima* (0.2, 1, or 5 mg/kg) or 0.9% saline solution + DMSO (vehicle). Experimental Periodonttis (EP) was performed as previously described (Bezerra et al., 2002). Briefly, the animals were anesthetized with ketamine and xylazine (90:10 mg/kg, i.p.) and a sterilized nylon suture thread (3.0 Nylpoint, Ceará, Brazil) was placed around the second left maxillary molar. To facilitate suture placement, a guide through the medial and distal interproximal spaces was made using a 5.0 suture needle (Point Suture, Ceará, Brazil). Groups received vehicle and *S. maritima* (*per os*) 1 h before periodontitis induction and once daily, during 11 days. After this time, blood samples were obtained and an overdose of ketamine and xylazine (300:30 mg/kg; i.p.) were used to euthanize the animals. The maxillae were removed to analyze the total bone resorption area. Hystopathological analyses was performed with another animals following the same above designed groups (*n* = 30).

Sm at a dose of 5 mg/kg was found to be the most effective dose against alveolar bone loss (ABL), and therefore this dose was selected for the experiments. Subsequent series of experiments were conducted with the following groups: non-ligated (naive - animals not subjected to EP); ligature only (vehicle), and ligature plus treatment with *S. maritima* 5 mg/kg diluted in 0.9% saline solution + DMSO (*S. maritima* 5) to quantification of gingival levels of cytokines TNF-α, IL-1β, CINC-1 and IL-10 (*n* = 18), antioxidant enzymes superoxide dismutase (SOD) and catalase (CAT) (*n* = 18) and qRT-PCR analysis of TNF-α, IL-1β, RANK, and iNOS (*n* = 18). All these assays were performed using the gingival tissues from surrounding maxillary left molars (the half of the maxillae with ligature).

### Measurement of alveolar bone loss

After the period of EP the maxillae were removed, divided in half and fixed in buffered formalin (10%) for 24 h. Following, the maxillae were defleshed and kept in 8% sodium hypochlorite for 4 h (Pimentel et al., 2012). After that, the specimens were washed in running water and stained with methylene blue (1%) to differentiate bone from teeth. Then, hemi-maxillae were fixed in a piece of wax with their occlusal planes parallel to the ground and long axes perpendicular to the camera and photographed with a 6.1-megapixel digital camera (Canon® 60D). ImageJ® software (National Institute of Health, Bethesda, MD, USA) were used for measurement of ABL, as described previously (Kuhr et al., 2004). The buccal area (mm^2^) corresponding to the exposed roots and coronary surface of the molars was calculated and obtained and subtracted from the correspondent area (mm^2^) of the normal right hemi-maxillae.

After definition of the most effective dose, one sample of each group was scanned using a high resolution microcomputed tomography (micro-CT) system (SkyScan 1174; Bruker-microCT, Kontich, Belgic, 50 kV and 800 μA) with a 0.5 mm aluminium filter and 15% beam hardening correction and ring artifacts reduction. We used 180 degrees of rotation and exposure range of 1 degree, time of scanning 38 min. Each specimen was scanned with acquisition of images every 0.7°, filed in TIFF format, with resolution of 19.7 μm and saved on a hard disk. After reconstruction of the images (NRecon v1.6.9; Bruker-microCT) 3D models were created with help of CTAn software v.1.12 program (Bruker-microCT) in accordance with the recommended guidelines.

### Histopathological analysis

The maxillae with ligature were fixed in 10% neutral-buffer formalin (10%) and demineralized in formic acid (10%). The specimens went through the process of dehydration, paraffin embedment, and after were sectioned in a mesio-distal plane for hematoxylin and eosin (HE) staining. The area between the first and second molars where ligatures had been placed was sectioned in 4 μm thickness sections and evaluated under light microscopy considering the inflammatoryparameters such as cell influx and alveolar bone and cementum integrity. Previously standardized scores ranging from 0 to 3 were used (Lima et al., 2000; Leitão et al., 2005). Score 0 indicates absence of or only discrete presence of inflammatory cell infiltration cellular infiltration (restricted to the marginal gingiva, preserved alveolar process and cementum; score 1 represents moderate inflammatory cellular infiltration all over the insert gingiva, some minor alveolar process resorption and intact cementum; score 2 represents accentuated cellular infiltration in both gingiva and periodontal ligament, accentuated degradation of the alveolar process, and partial destruction of cementum; score 3 indicates accentuated cellular infiltration with complete resorption of the alveolar process and intense destruction of cementum. Two examiners who were masked to the identity of samples performed histologic evaluation.

### Plasma bone alkaline phosphatase (BALP)

The plasma concentration of bone alkaline phosphatase was obtained from blood samples collected from the retro-orbital plexus on the 11th day using the thermo-activation method, as previously described (Whitby and Moss, 1975). The samples were heated up to 56°C for 10 min. Serum levels of BALP were calculated by subtracting the concentration of the heated alkaline phosphatase in serum from the concentration of the total alkaline phosphatase (TALP) in serum. The analysis was performed according to the manufacturer's instructions (Labest, Lagoa Santa, MG, Brazil).

### Quantification of cytokine levels in gingival tissue

The stored gingival tissues were used to determine the concentrations of TNF-α, IL-1β, CINC-1 and IL-10 using specific commercially available kits (DuoSet Elisa kit, R&D Systems Inc., MN, USA). The inflammatory mediators' levels were determined by enzyme-linked immunosorbent assay (ELISA) using respective standard curves. Results were shown as picogram/ml (pg/ml). All kits were used according manufacturer's instructions.

### Superoxide dismutase (SOD) and catalase (CAT) levels in gingival tissue

The gingivae removed on 11th day were used to evaluate the effect of *S. maritima* 5 mg/kg on the oxidative stress. The SOD activity was assayed according the protocol previously described (Beauchamp and Fridovich, 1971). In a dark room, the gingival samples were homogenized in 20 μl of ice-cold phosphate buffer at 15,000 G for 20 min. The supernatants were mixed with a solution comprised of phosphate buffer (50 nM), EDTA (100 nM) and L-methionine (19.5 mM) in a pH of 7.8. Then, 150 ml of a solution of riboflavin (10 nM) and nitro NBT (750 nM) was added and the mixture was exposed to light (20 W) for 15 min. The absorbance of the samples was measured at 560 nm. The results are expressed as grams of SOD per ml.

The measurement of O_2_ production rate and H_2_O in proportion of H_2_O_2_ was calculated to obtain CAT activity. Briefly, 20 μl of gingival homogenate was mixed with a solution comprised of 3% H_2_O_2_ and Tris-HCl EDTA buffer (5 nM, pH 8.0). The absorbance was measure immediately and 6 min after preparing the samples at a 230 nm wavelength (Maehly and Chance, 1954).

### qRT-PCR analysis of TNF-α, IL-1β, RANK, and iNOS levels

Total RNA was extracted from gingivae tissues using the TRIzol reagent (Invitrogen, São Paulo, Brazil). The RNA concentration was estimated by reading the absorbance at 260 nm and was checked for purity at 280 nm in a spectrophotometer (Amersham Biosciences, Cambridge, England). For each sample, the RNA concentrations used to synthesize cDNA were adjusted to 1,000 ng/mL. Before the reverse transcription reaction, samples of RNA were incubated for 5 min at 70°C and then cooled in ice. The reverse transcription was performed in a total volume of 20 μL composed of 10 μL of sample RNA, 4 μL reverse transcriptase buffer (Invitrogen), 8 units RNAsin, 150 units of reverse transcriptase Superscript III, 0.036U random primers, 10 mM DTT and 0.5 mM of each dNTP (Invitrogen). The mixture was incubated at 42°C for 1 h, subsequently at 80°C for 5 min and finally stored at −20°C. The negative control was prepared under the same conditions but without addition of reverse transcriptase.

Quantitative real-time polymerase chain reaction (qRT-PCR) was performed in triplicate to determine the gingival levels of mRNA for TNF-α, IL1-β, RANK and iNOS. Each reaction contained 10 μL of SYBR® Green Master Mix (Applied Biosystems, Warrington, UK), 7,3 μL of ultra-pure water, 1 μL of cDNA and 0.5 μM of each primer and was performed in StepOne Real-Time PCR (Applied Biosystems, Warrington, UK) thermocycler. The thermal cycling profile for the first round of qRT-PCR was initial denaturation and activation of the polymerase for 10 min at 95°C, followed by 40 cycles of 15 s at 95°C, 30 s at 58°C, and 30 s at 72°C. The final extension was for 10 min at 72°C. The primers were designed by using the PrimerQuest® Tool https://www.idtdna.com/Primerquest/Home/Index. The primers used in this study are shown in Table 1. The glyceraldehyde 3-phosphate dehydrogenase (GAPDH) was used as endogenous control for normalization of messenger RNA expression. The specificity of each primer pair was confirmed by melting curve analysis of qRT-PCR products. Relative quantifications of mRNA were carried out using the comparative threshold (CT) cycle method. The delta-delta-Ct method was used to transform the Ct values into normalized relative expression levels (Livak and Schmittgen, 2001).

**Table 1:** Description of biomarkers, gene, primer sequences and NCBI accession numbers.

**Biomarkers**	**Gene**	**Primer sequence (5′-3′)**	**NCBI**
Inflammatory modulators	TNF-α	**S-**CGGGGTGATCGGTCCCAACAAG	NM_012675.3
		**A-**GTGGTTTGCTACGACGTGGGC	
	IL-1β	**S-**TGCTGTCTGACCCATGTGAG	NM_031512.2
		**A-**CCAAGGCCACAGGGATTTTG	
	iNOS	**S-**AGGCACAAGACTCTGACACC	NM_012611.3
		**A-**GGTAGGGTAGAGGAGGGGAG	
Bone makers	RANK	**S-**AGGGAAAACGCTGACAGCTAA	NM_001271235.1
		**A-**CCAACACAATGGTCCCCTGA	
Reference gene	GAPDH	**S-**GGACCAGGTTGTCTCCTGTG	NM_017008.4
		**A-**CATTGAGAGCAATGCCAGCC	

### Subchronic toxicity evaluation

Variation of body mass, organ weight alteration and biochemical and histopathological parameters were evaluated in animals treated daily during 11 consecutive days with a single dose of *S. maritima* 5 mg/kg and saline + DMSO. Peripheral blood samples were collected in order to obtain biochemical analysis of aspartate aminotransferase (AST) and alanine aminotransferase (ALT) levels and creatinine (Labtest, Lagoa Santa, MG, Brazil).

After sacrificing the animals, the liver, kidney and heart were removed and weighed. The stomach was also removed for histological analysis. The organs were fixed with formalin and then dehydrated with increasing concentrations of ethanol and embedded in paraffin. The blocs were sliced in 5 μm thick sections, stained with hematoxylin-eosin (HE) and observed at light microscope (Leica DM 2000, Wetzlar, Germany).

### Statistical analysis

Shapiro-Wilk normality test was performed to analyze the data. Results are presented as means ± standard error (SEM) or as medians when appropriate. ANOVA followed by Tukey test or Games-Howell test were used to compare means and Kruskal–Wallis and Dunn tests were used to compare medians. *P* < 0.05 was considered significant. Analyses were performed using IBM SPSS Statistics for Windows, Version 20.0. Armonk, NY or GraphPad Prism 6 software, San Diego, CA, USA.

## Results

### Alveolar bone loss and histopathological analysis

**Figure 1:**
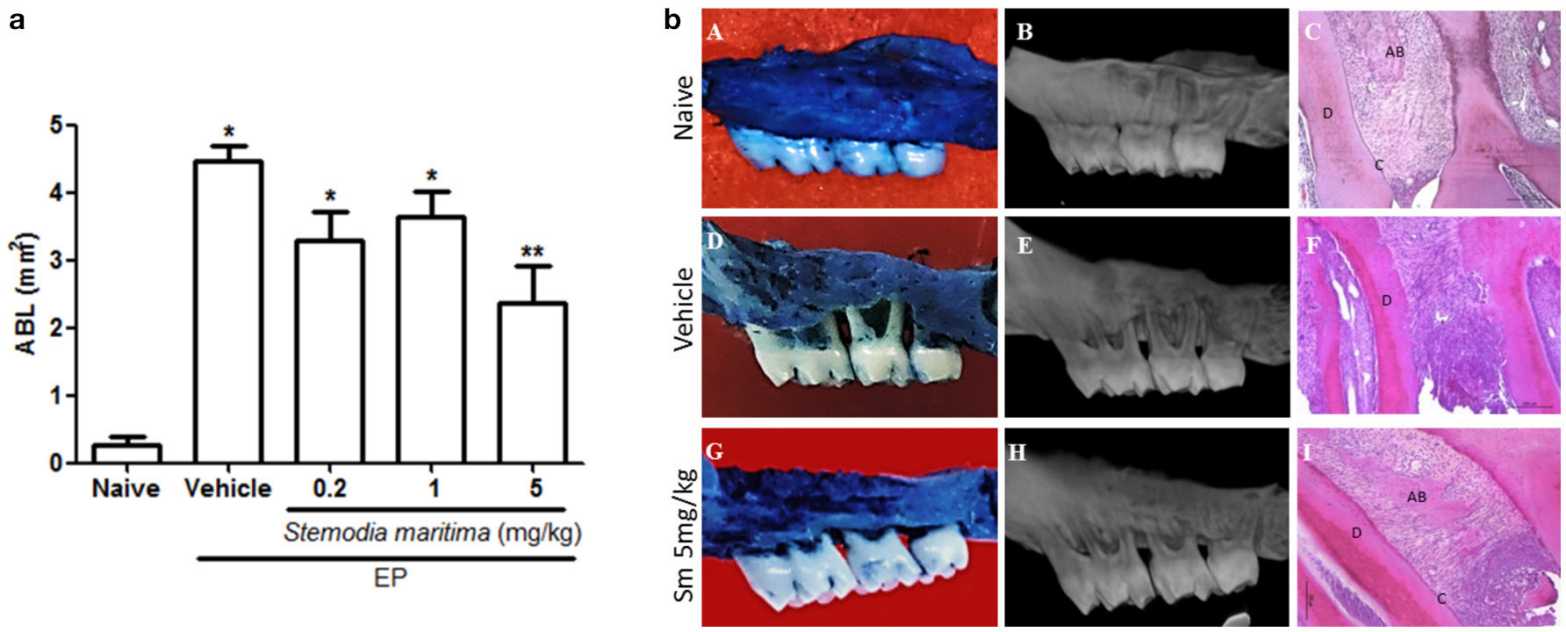
**(a)** Effect of oral gavage of vehicle (saline + DMSO) and Sm extracts concentrations on alveolar bone loss (0.2, 1, and 5 mg/kg) in experimental periodontitis. Data represent the mean ± SEM of six animals/group. ^*^P < 0.05 was considered significantly different compared to the naïve control group; ^**^P < 0.05 was considered significantly different compared to vehicle group (saline + DMSO). (ANOVA and Games-Howell *post-hoc* test). **(b)** Macroscopic view (first column), microCT images (second column) and histological aspects (third column) of Naive, Vehicle and Sm 5 mg/kg. Data represent the mean ± SEM of six animals/group. **(A–C)** Indicates normal maxilla (naive), showing integrity of its components (c, Cementum; d, Dentine and ab, Alveolar bone). **(D–F)** Shows maxilla subjected to experimental periodontitis that received only the vehicle (saline + DMSO), showing severe bone resorption, inflammatory infiltrate in gingiva and periodontal ligament, extensive cementum destruction and total resorption of the alveolar process. **(G–I)** Indicate maxilla after 11 days of experimental periodontitis treated with Sm 5 mg/kg showing discrete cell influx and preservation of the alveolar process and cementum. Magnification x100.

No significant ABL was observed in the naive group (Figures 1a,b). Data indicated that *S. maritima* 5 mg/kg was the most effective dose protecting against ABL. Gavage (*per os*) administration of *S. maritima* 5 mg/kg 1 h before the placement of the ligature and once daily for 11 days resulted in a significant (*P* < 0.05) inhibition of ABL (2.37 ± 0.53) (Figures 1a,bG,H), compared to the group that received the vehicle only (4.47 ± 0.22) (Figures 1a,bD,E).

Histopathologic analysis of animals subjected to EP (vehicle group) demonstrated accentuated inflammatory cell infiltration, breakdown of alveolar bone, collagen fiber derangement within the periodontal ligament, and resorption of cementum, receiving a median score of 3 (range 2 to 3) (Table 2). The periodontium of animals treated with *S. maritima* 5 mg/kg showed preservation of the alveolar process and cementum, reduction of the inflammatory cell infiltration, and partial preservation of collagen fibers of the periodontal ligament (Figures 1bC,F,I), receiving a median score of 1 (range 1 to 2) (Table 2). These values were statistically different (*P* < 0.05), compared with the vehicle group.

**Table 2:** Effect of oral gavage of Sm extracts and vehicle (saline + DMSO) on histopathologic score of rat maxillae.

			***Stemodia mar**í**tima*** **(Sm) mg/kg**		
	**Naive**	**Vehicle**	**0.2**	**1**	**5**
Escores (median values and range)	0 (0–0)	3 (2–3)^*^	2 (1–2)	2 (2–2)	1 (1–2)^**^

* *P < 0.05 vs. naïve group (CONTROL)*;

** *P < 0.05 vs. vehicle group (animals submitted to experimental periodontitis and treated with saline + DMSO) (Kruskal-Wallis followed by Dunn's test)*.

### Plasma bone-specific alkaline phosphatase (BALP)

**Figure 2:**
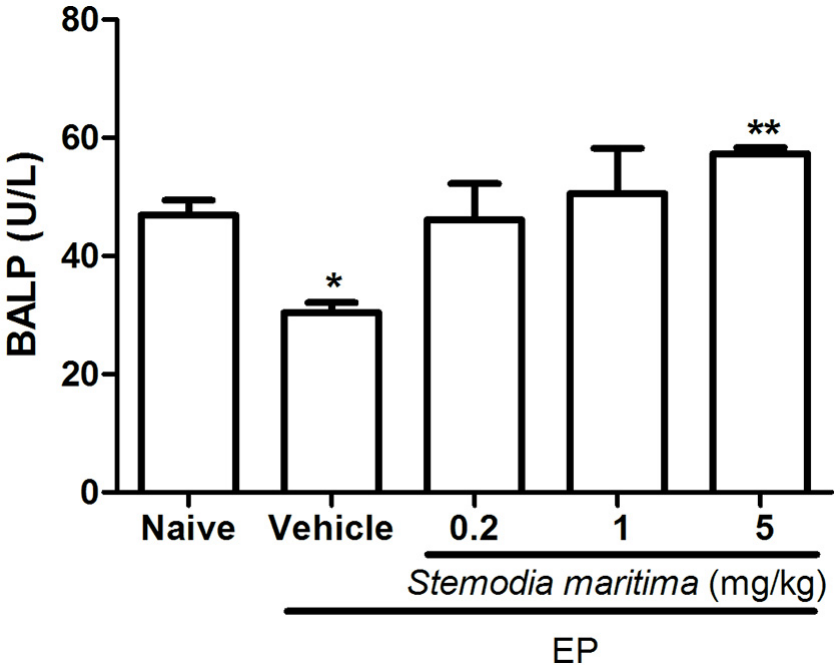
Effects of Sm 5 mg/kg and saline + DMSO (vehicle) on the plasma bone alkaline phosphatase (BALP) in experimental periodontitis in rats. Data represent the mean ± SEM of six animals/group. ^*^P < 0.05 compared with naïve group (control); ^**^P < 0.05 compared with vehicle group (animals submitted to experimental periodontitis and treated with saline + DMSO) (ANOVA and Games-Howell *post-hoc* test).

Experimental periodontitis decreased BALP serum levels and the treatment with *S. maritima* 5 mg/kg resulted in a significant increase in the BALP serum levels (*P* < 0.05), when compared with the vehicle group (Figure 2).

### Effects of *S. maritima* on cytokine levels in gingival tissue

**Figure 3:**
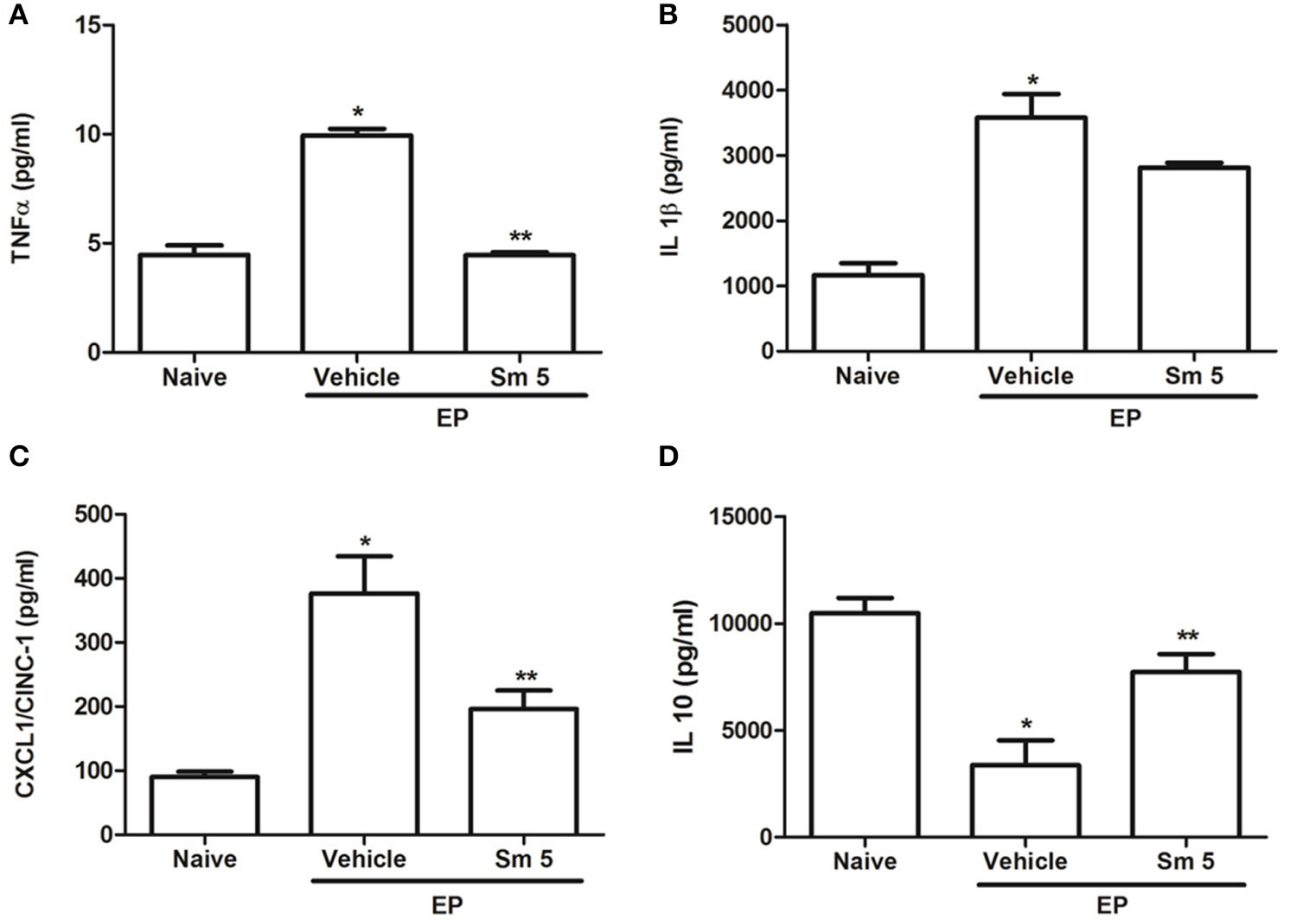
Effects of Sm 5 mg/kg and saline + DMSO (vehicle) on TNF-α **(A)**, IL-1β **(B)**, CINC-1 **(C)** and IL-10 **(D)** in gingival tissues levels. Data represent the mean ± SEM of six animals/group. ^*^P < 0.05 compared with naïve group (control); ^**^P < 0.05 compared with vehicle group (animals submitted to experimental periodontitis and treated with saline + DMSO) (ANOVA and Tukey's *post-hoc* test).

Periodontitis challenge was associated with significant (*P* < 0.05) increase of pro-inflammatory cytokines (TNF-α, IL-1β, and CINC-1) in gingival tissue; at the same time it was observed a significant (*P* < 0.05) decrease in gingival levels of IL-10, an anti-inflammatory cytokine, when compared to naive group. Treatment with Sm 5 mg/kg significantly (*P* < 0.05) decreased TNF-α and CINC-1 but no significant difference was observed in IL-1β gingival levels; also, *S. maritima* 5 mg/kg increased IL-10 levels, when compared to vehicle treated group (Figures 3A–D).

### Superoxide dismutase and catalase levels in gingival tissue

**Figure 4:**
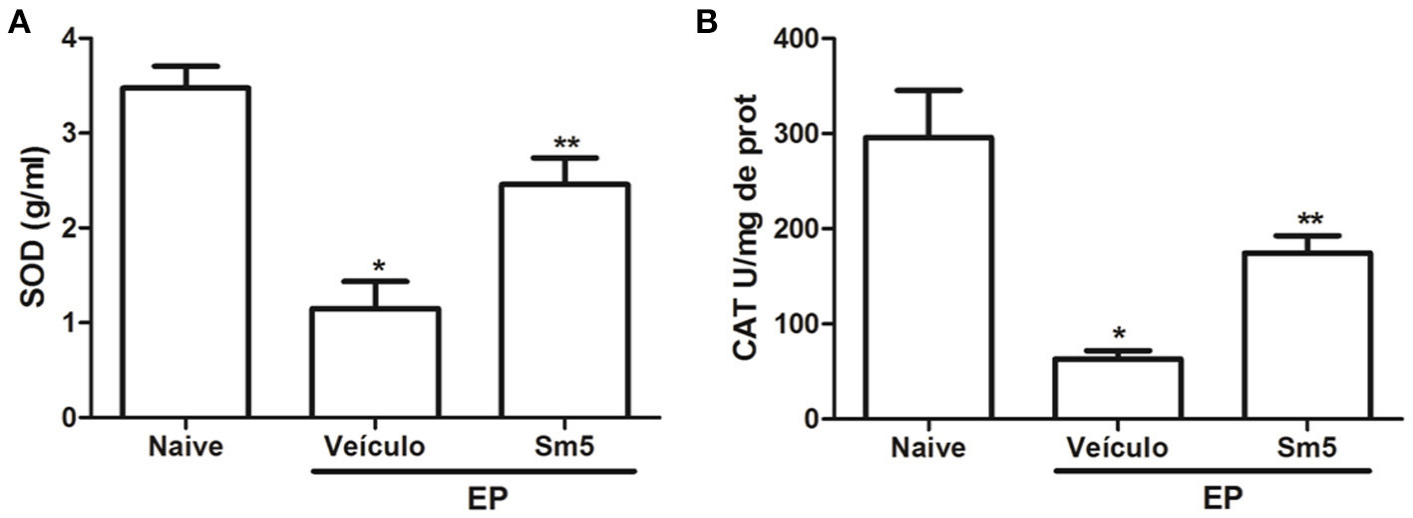
Effects of Sm 5 mg/kg on superoxide dismutase **(A)** and catalase **(B)** concentrations in gingival tissues. Data represent the mean ± SEM of six animals/group. ^*^P < 0.05 compared with naïve group (control); ^**^P < 0.05 compared with vehicle group (animals submitted to experimental periodontitis and treated with saline + DMSO) (ANOVA and Tukey's *post-hoc* test).

In the present study periodontitis induction resulted in a significant (*P* < 0.05) reduction of SOD and CAT levels in gingival tissue, when compared with naive group. *S. maritima* 5 mg/kg significantly (*P* < 0.05) increased both markers for oxidative stress in gingival tissue, when compared with vehicle group (Figures 4A,B).

### qRT-PCR analysis of TNF-α, IL-1β, RANK and iNOS levels

**Figure 5:**
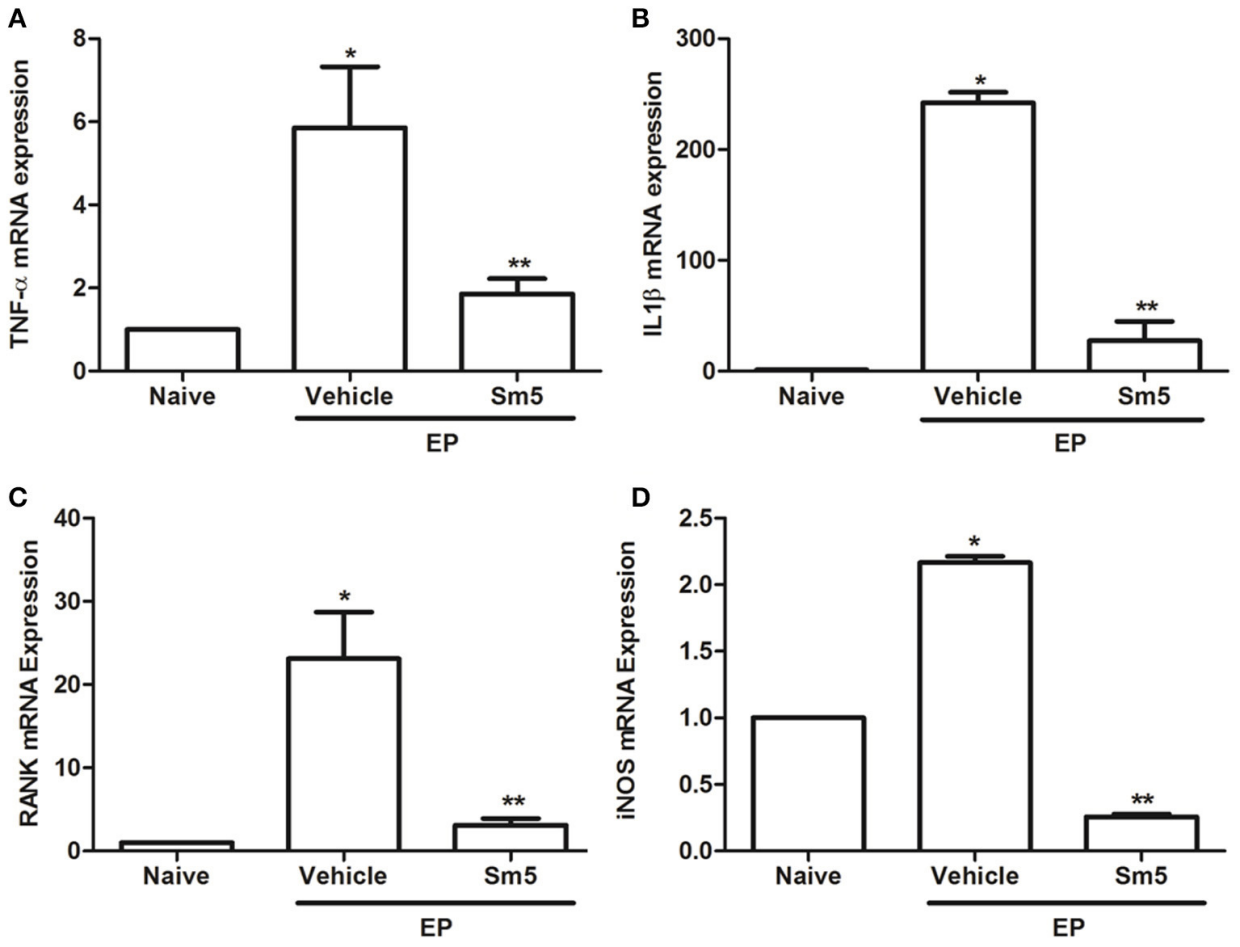
Effects of Sm 5 mg/kg on tumor necrosis factor alpha (TNF-α) **(A)**, interleukin 1β (IL-1β) **(B)**, RANK **(C)**, and inducible nitric oxide synthase (iNOS) **(D)** mRNA levels in experimental periodontitis in rats. Data represent the mean ± SEM. ^*^P < 0.05 compared with naïve group (control); ^**^P < 0.05 compared with vehicle group (animals submitted to experimental periodontitis and treated with saline + DMSO) (ANOVA and Tukey's *post-hoc* test).

The vehicle groups showed significant increase in TNF-α (Figure 5A), IL-1β (Figure 5B), RANK (Figure 5C) and iNOS (Figure 5D) mRNA expression, when compared with the naive control group. Administration of *S. maritima* 5 mg/kg significantly reduced mRNA expression in all parameters evaluated compared to vehicle group (Figure 5).

### Analysis of toxicity

No signs of systemic illness, adverse pharmacological events or changes in behavior were observed throughout the experimental period. *S. maritima* 5 mg/kg or vehicle did not affect the animals' body mass or the wet weight of the livers, kidneys or hearts compared to the naive control throughout the study period. Gross necropsy findings did not show any abnormalities. A single-dose of *S. maritima* (5 mg/kg) or vehicle (saline + DMSO) over 11 consecutive days had no significant histological alterations in the hepatic, renal parenchyma, and cardiac tissue. The values obtained for ALT/AST and creatinine did not differ from the control group.

## Discussion

Ligature-induced periodontitis is a well-established animal model. While it has some limitations, the structure and organization of the periodontal tissues are similar to those of humans. Further, rat models have fewer limitations than *in vitro* models that cannot reproduce the complexity of interactions among the oral microbiome, environmental factors, and the immune/inflammatory host response (Struillou et al., 2010; Oz and Puleo, 2011; Hajishengallis et al., 2015). In this model, ligature causes mechanical trauma, and affects tissue integrity to induce an inflammatory response, increase oxidative stress, and destroy the periodontal ligament leading to significant bone loss (Bezerra et al., 2000; Guimarães et al., 2016; Lima et al., 2017; Oliveira et al., 2017). Alternative and preventive treatment options are essential to overcome the adverse effects of both antimicrobial and anti-inflammatory agents as an adjunct to conventional mechanical and surgical treatments (Chandra Shekar et al., 2015; Ramesh et al., 2016).

Chemical studies on *S. maritima* reported the isolation of diterpenes and flavonoids showing antiviral, cytotoxic, and larvicidal activities (Hufford et al., 1991; Rodrigues et al., 2010). Further, it was showed that *S. maritima* compounds have *in vitro* inhibitory activity of both lipids peroxidation and cyclooxygenase 1 and 2 (Hufford et al., 1992; Russell et al., 2011). Besides, our research group demonstrated that *S. maritima* has antioxidant activity *in vitro* and it also has activity against some bacterial strains (da Silva et al., 2014). However, it is important to mention that there is no study in the literature using *S. maritima* extract or its identified compounds on bone resorption models. These data encouraged us to investigate whether *S. maritima* extract could be useful to ameliorate the bone loss during periodontitis.

The protective effect of *S. maritima* on alveolar bone loss was associated with an increase in plasma bone-specific alkaline phosphatase (BALP) suggesting that 5 mg/kg *S. maritima* prevents bone resorption and stimulates bone formation. Many studies have verified the effects of medicinal plants on ABL using a similar periodontitis model (Sezer et al., 2013; Hatipoglu et al., 2015; Saglam et al., 2015; Guimarães et al., 2016). Sezer et al. (2013) showed that the systemic use of *Ginko biloba* extract on reducing ABL. Hatipoglu et al. (2015) assessed ABL from microcomputed tomography (micro CT) images, and verified less bone resorption in animals treated with *Crataegus orientalis* extracts. Recently, Guimarães et al. (2016) observed the ability of the *Matricaria recutita* extract to inhibit TNF-α and IL-1β cytokines. These treatments prevented the osteoclast activation via RANKL-OPG. Lima et al. (2017) demonstrated anti-inflammatory and anti-oxidant activities with *Calendula officinalis*.

Cytokines play a significant role in periodontitis. Here, we showed that *S. maritima* (5 mg/kg) significantly increased IL-10 gingival levels, an anti-inflammatory cytokine, while decreasing the pro-inflammatory cytokines TNF-α and CINC-1. Alternative therapeutic approaches based that inhibit TNF-α production have been successfully used for the pre-clinical and clinical treatment of chronic inflammatory diseases, particularly rheumatoid arthritis and temporomandibular joint disorders (Feldmann, 2002; Araújo et al., 2013; Freitas et al., 2016; Alves et al., 2017). Further, when mRNA expression for TNF-α, IL-1β, iNOS, and RANK were evaluated, *S. maritima* 5 mg/kg significantly reduced mRNA expression for all these genes compared to the vehicle group. Cytokines amplify the inflammatory response in periodontitis (Duarte et al., 2015). It seems contradictory that *S. maritima* 5 mg/kg reduced mRNA expression for IL-1β without affecting IL-1β gingival levels. Here, we hypothesized that the IL1-β levels detected by ELISA in gingival samples might derive from a pre-formed pool because the increase in IL-1β levels after periodontitis challenge precedes the increase in mRNA. Studies analyzing the effects of resveratrol, a naturally occurring product found in numerous plants showed similar results (Casati et al., 2013; Tamaki et al., 2014).

The release of large amounts of NO by iNOS plays a major role in immune-inflammatory events including periodontitis (Leitão et al., 2005; Tamaki et al., 2014; Martins et al., 2016). TNF-α and IL-1 trigger the transcription of the iNOS resulting in the increase production of NO. On the other hand, the significant increase of IL-10 levels in gingival tissues could also decrease iNOS gene expression (Gadek-Michalska et al., 2013). Some components of medicinal plants might inhibit nuclear transcription factor-kB binding activity and downregulate the expression of iNOS (Kim et al., 2005; Cai et al., 2008). These results are in accordance with our present data since the extract of *S. maritima* decreased transcription of iNOS genes.

Oxidative stress plays a central role in periodontitis (Tóthová et al., 2015; Lima et al., 2017). This study demonstrated that experimental periodontitis in rats leads to oxidative stress as indicated by a significant reduction in SOD and CAT levels in gingival tissue. This effect was reduced in animals treated with *S. maritima* (5 mg/kg), which suggests that the beneficial effects of *S. maritima* are at least partially related to its antioxidative properties. *Calendula offinalis* extract also presents ability to reduce oxidative stress by increasing superoxide dismutase (SOD), catalase (CAT) and reduced gluthatione (GSH) enzymes and decreasing malonaldeyde (MDA) levels in gingival tissue (Lima et al., 2017).

The regulation of the critical cytokine macrophage colony-stimulating factor, RANK ligand is essential to the differentiation of osteoclasts. Our results indicated that ligature-induced periodontitis in rats is associated with an increase in the RANK mRNA levels in periodontal tissues, and the treatment with *S. maritima* (5 m/kg) could reduce RANK expression.

Medicinal plants have been historically used for treatment of numerous human diseases (Varela-López et al., 2015; de Oliveira et al., 2016; Ramesh et al., 2016). The leaves and stem of *S. maritima* can treat stomach pain and fluid retention, although the literature still lacks studies that confirm the safety of this plant. Thus, in this study we used biochemical and histopathological analysis to show that the administration of *S. maritima* (5 mg/kg) did not promote any signs of toxicity when administered for 11 consecutive days.

This study observed that 5 mg/kg *S. maritima* extract reduced alveolar bone loss, inflammation, and oxidative stress in a ligature-induced model of periodontitis in rats without causing any systemic changes. These data are in accordance with previous results from our group, which demonstrated via *in vitro* assays that *S. maritima* has anti-inflammatory, antioxidant and antibacterial properties (da Silva et al., 2014). We also found evidence that, at least in part, the effectiveness of *S. maritima* depends upon a positive balance between pro and anti-inflammatory cytokines that decrease TNF-α, IL-1β, and CINC-1 gingival levels while increasing IL-10. This effect might also modulate both iNOS activity and RANK levels, to improve antioxidative events.

To the best of our knowledge, there are no existing reports evaluating the efficacy of *S. maritima* extract in a preclinical trial of rat periodontitis. Although additional studies are needed, these data suggest that *S. maritima* is a potential tool for controlling the development of periodontitis.

## Author contributions

AT and JF treated the animals, performed all assays, analysis and interpretation of data. LdS induced periodontitis. AA and FL performed the collection and extraction of plant material. NdS performed cytokines analysis. IM, KP, and IC performed histolopathogical analysis. AP assisted in laboratory experiments. JC performed quantitative qRT-PCR analysis. GC-F and VP performed and analyzed the *S. maritima* Linn toxicity profile. MB, HC, and PG designed and supervised the study. AT and MB wrote the paper. All authors critically reviewed and approved the manuscript.

### Conflict of interest statement

The authors declare that the research was conducted in the absence of any commercial or financial relationships that could be construed as a potential conflict of interest.

## Abbreviations

cDNA: Complementary Deoxyribonucleic Acid
CINC-1: Rat cytokine-induced neutrophil chemoattractant – 1
DMSO: Dimethyl sulfoxide
dNTP: Deoxynucleotide
DTT: Dithiothreitol
EDTA: Ethylenediaminetetraacetic acid
HE: Hematoxylin and eosin
IL-10: Interleukin 10
IL1-β: Interleukin 1 beta
NBT: Nitro-blue tetrazolium
qRT-PCR: Reverse Transcription Polymerase Chain Reaction
RANK: Receptor activator of nuclear factor-kappaB
RNA: Ribonucleic Acid
TNF-α: Tumor Necrosis Factor-alpha.
